# Cigarette Smoke, Bacteria, Mold, Microbial Toxins, and Chronic Lung Inflammation

**DOI:** 10.1155/2011/819129

**Published:** 2011-07-09

**Authors:** John L. Pauly, Geraldine Paszkiewicz

**Affiliations:** Department of Immunology, Roswell Park Cancer Institute, Elm and Carlton Streets, Buffalo, NY 14263, USA

## Abstract

Chronic inflammation associated with cigarette smoke fosters malignant transformation and tumor cell proliferation and promotes certain nonneoplastic pulmonary diseases. The question arises as to whether chronic inflammation and/or colonization of the airway can be attributed, at least in part, to tobacco-associated microbes (bacteria, fungi, and spores) and/or microbial toxins (endotoxins and mycotoxins) in tobacco. To address this question, a literature search of documents in various databases was performed. The databases included PubMed, Legacy Tobacco Documents Library, and US Patents. This investigation documents that tobacco companies have identified and quantified bacteria, fungi, and microbial toxins at harvest, throughout fermentation, and during storage. Also characterized was the microbial flora of diverse smoking and smokeless tobacco articles. Evidence-based health concerns expressed in investigations of microbes and microbial toxins in cigarettes, cigarette smoke, and smokeless tobacco products are reasonable; they warrant review by regulatory authorities and, if necessary, additional investigation to address scientific gaps.

## 1. Introduction: Chemical and Biological Components of Tobacco and Smoke

For many years, scientists have undertaken studies to define the chemical composition of green tobacco leaf, cured-fermented-stored tobacco leaf, and tobacco smoke with the intent of identifying chemicals that may pose a significant health risk [1–4]. An illustration has been prepared of the annual increase, from 1954 to 2005, in the total number of tobacco smoke chemicals that have been identified [4]. Today, there is a consensus of opinion that cigarette smoke consists of at least 5,300 different chemicals [4]. These chemicals are present in the complex aerosol that consists of a heterogeneous mixture of gas- (vapor-) phase and particulate- (“tar-”) phase components [1–4]. 

Detailed listings of the chemicals in mainstream and sidestream tobacco smoke are available, and an assessment of their propensity for harm has been presented; a partial listing of references is included [1–4]. Most of the chemicals, toxicants, and carcinogens in tobacco smoke arise from the burning (pyrolysis) of the tobacco [1, 2, 4]. The potential for harm has also been studied for chemicals that do not arise from the burning of tobacco. The chemicals include metallic and nonmetallic elements, isotopes, and salts [1, 2, 4]. In addition, pesticides and other intact agrochemicals have been identified in tobacco smoke [1, 2, 4]. Also included in this tabulation of chemicals in smoke are menthol and flavorants [4]. 

In 1985, Hoffmann and coworkers, who had studied the chemical composition of tobacco smoke for many years, began formulating a list of chemicals that were designated as biologically active, carcinogenic, cocarcinogenic, or tumorgenic, reviewed previously in [4]. The tabulation was revised and became the basis for the list of “*Hoffmann Analytes*” [4]. In 1985, different working groups met to identify those chemicals in tobacco smoke that are most likely to be carcinogenic to humans as defined by criteria of the International Association for Research on Cancer (IARC), an intergovernmental agency forming part of the World Health Organization, and by the US National Toxicology Program (NTP) [1, 2, 4]. 

## 2. The Changing Cigarette

The identification, classification, and concentration of the various chemicals in cigarette smoke have been challenged by changes in the design of cigarettes. A comprehensive review of “The Changing Cigarette” was published by D. Hoffmann and I. Hoffmann in 1997 [5]. 

Subsequently, other investigators addressed changes in cigarettes and their potential for risk [6–12]. By way of example, a partial tabulation of changes in cigarette includes (a) increased cigarette length (85 mm king sized and extra long “120's”) and, for some brands, reduced circumference (23 mm “slim” cigarettes), (b) variation in the blend of natural tobaccos of diverse types, country of origin, and curing processes, relative percent tobacco leaf (lamina) versus tobacco ribs/stems, and tobacco weight per rod, (c) incorporation of manmade tobacco, sometimes referred to as reconstituted or “sheet” tobacco, (d) introduction of additives to the tobacco (casings) that include diverse flavorings (licorice and honey), humectants to retain tobacco moisture, and menthol to ameliorate smoke irritation and promote smoking acceptance by youngsters and “starters” (e) addition of ammonia, to facilitate “freebasing” the nicotine to enhance the pharmacological effect (impact), (f) application of diverse glues and printing ink, (g) configuration of diverse cigarette filter materials (cellulose acetate, paper, or combination of both), (h) alteration of filters with charcoal and schemes whether the carbon was dispersed throughout the filter plug or retained in a filter cavity, (i) variation in filter design (filter length, fiber packing/crimping, fiber density, and filter ventilation) to effect tar delivery (full flavor cigarettes versus ultralight low-tar cigarettes), (j) paper type, paper porosity, with burn accelerators to promote burning, or with modifications to reduce the propensity for sustained burning and affect a “fire safe” designation, and (k) diverse methodologies to reduce “tar” and nicotine yields in mainstream smoke of cigarettes that have been smoked mechanically [6–12]. 

The topic of  “*The Changing Cigarette*” has been addressed and summarized in a recent report of the Surgeon General entitled “How Tobacco Smoke Causes Disease” [13]. A review of the scientific and medical literature has shown that (a) changing cigarette designs over the last five decades, including the introduction of cigarette filters and low-tar cigarettes, have not reduced overall disease risk among smokers and may have hindered prevention and cessation efforts, (b) there is insufficient evidence that novel tobacco products reduce individual and population health risks, and (c) the introduction of novel tobacco products that are marketed as reduced-risk cigarettes may encourage tobacco use among youngsters. These changes have challenged tobacco policy and regulation [13].

## 3. Tobacco and Harm Associated with Microbes

 Our review of the aforementioned writings [1–4] and many other related reports, addressing chemicals in tobacco smoke of cigarettes have shown that the writings do not address the propensity for harm that may be associated with microbial elements of smokeless and smoking tobacco articles. A partial listing of tobacco-associated microbial elements include bacteria (Gram positive and Gram negative), bacterial spores, fungi (yeast and mold), fungal spores, cell wall components (certain glucans and flagellum), and diverse microbial toxins that include exotoxins and endotoxins. Examples of bacterial-derived toxins include endotoxins (lipopolysaccharide, LPS; inflammatory factor) and fungal-derived mycotoxins (aflatoxins, AF type B1; human carcinogen) [1–4]. 

There exists today a concern of the potential health risks associated with diverse microbial elements that are known to exist in smoking and smokeless tobacco products that are currently being marketed. This subject has not been addressed in the context of national tobacco control policy or regulatory authorities. 

**Figure 1:**
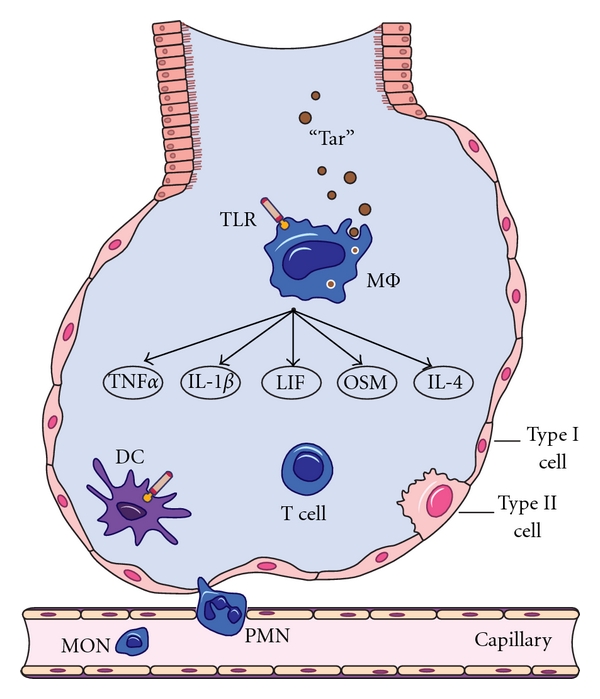
A schematic view of an alveolus that depicts the effect of inhaled tobacco smoke on the terminal (respiratory) structure of the lung. Particulate matter “Tar” in tobacco smoke is inhaled deep into the lung where it is recognized by macrophages. The macrophages arise from the blood monocytes that migrate into the lung where they undergo differentiation and maturation. Macrophage phagocytosis of the chemical-rich “Tar” evokes the production of diverse proinflammatory mediators (for details, see Figure 1). Macrophages have toll-like receptors (TLR) that recognize diverse microbes and toxins (LPS is recognized by TLR-4). Shown in this illustration is the production of five proinflammatory cytokines: tumor necrosis factor, type alpha (TNF*α*), interleukin 1-beta (IL-1*β*), leukemia inhibitory factor (LIF), oncostatin M (OSM), and Interleukin-4 (IL-4). These soluble factors interact with other cells of the lung, and the response of these cells is thought to accelerate, amplify, and prolong pulmonary inflammation. The target cells may include T cells. The T cell that is depicted herein is representative of many different T cell subsets, including T helper cell subsets Th1, Th2, and Th17. Type I epithelial cells are the major cells lining the alveolar space, and facilitating O_2_/CO_2_. The type I cells are spread out and cover about 90 to 95% of the alveolar surface. The type II cells form only 5 to 10% of the surface but produce surfactant proteins. Polymorphonuclear leukocytes (PMN) mediate inflammation in multiple ways, including the production of an oxidative burst. Dendritic cells (DC) are professional antigen-presenting cells; they also mediate inflammation.

Harm is to be recognized as persistent or chronic inflammation. Inflammation is mediated by different leukocyte subsets and different secreted factors (Figure 1). Inflammation not only establishes a microenvironment that fosters the malignant transformation and tumor growth but also promotes microbial colonization.

## 4. Research Objectives

The goal of this paper is to profile the scientific and medical literature addressing microbes in tobacco with the intent to determine whether there is sufficient evidence to warrant additional investigations to assess propensity for human harm. The impetus for undertaking this work was derived in part from the fact that several teams of investigators, including our own, have published observations during the last few years that suggest microbial elements maybe harmful to tobacco users. 

Notable in a first analysis of the literature on the microbiology of tobacco we discovered that there were few recent reports (1990 to 2010) in peer-reviewed, mainstream, scientific and medical journals by scientists of tobacco companies. By way of example, Philip Morris has contracted the Life Science Research Office, Inc., (LSRO, Bethesda, MD), to identify methods to evaluate tobacco products and with a particular focus on identifying research schemes and assays for assessing reduced-risk tobacco articles [14]. Three monographs published by LSRO in 2007 detailed the chemicals to be assayed and recommended procedures. The subject of microbial flora and microbial toxins was not addressed, nor were schemes and methodologies for the assessment of tobacco associated bacteria, mold, or microbial toxins [14]. 

Therefore, the question arose as to whether the issue of health risks associated with microbial elements in smokeless and smoking tobacco was not investigated by laboratory scientists working at the tobacco companies or whether the subject was studied and the information withheld as private and confidential. The paucity of the literature on health risks associated with microbes in smokeless and smoking tobacco is to be contrasted to the numerous reports by tobacco scientists researching other health-related issues, such as potential reduced-risk exposure tobacco products (PREPS) [15].

## 5. Perspective and Limitations

The authors are immunologists and have an active research interest in addressing tobacco-associated chronic pulmonary inflammation. It is acknowledged that immunological responses and inflammation would not be a primary interest by other investigators whose primary interests are in the disciplines of microbiology/metagenomics, aerosol-associated inhalation toxicology, infectious diseases, and clinical pathology (oral and lung). Also, the work presented herein is limited in scope. The authors retrieved numerous documents from databases, but space restrictions permit citing but a few of the writings. Also, many of the writings were internal documents and were not subjected to peer-review. Some documents cited are old and are addressed herein to provide a historical perspective. Lastly, the documents are fragmented and it is recognized that conflicting findings and interpretations may be presented by competing tobacco companies.

## 6. Literature Search

A computer-based structured search of the literature was conducted. The study scheme included a search of the literature from PubMed (http://www.ncbi.nlm.nih.gov/pubmed) and Scopus (http://www.scopus.com/home.url). Also, included was a search of Google (http://www.google.com/). A search was also made of patents in the database of the US Patent and Trade Office (http://www.uspto.gov/). In addition, searches were made for documents that were released by the tobacco companies and made public as a consequence of the tobacco Master Settlement Agreement. To this end, we searched database records of over 11 million documents in the digital archive that were established and which are maintained currently at the University of California, San Francisco (http://legacy.library.ucsf.edu/). We also searched the database from Tobacco Documents (http://tobaccodocuments.org/). 

The searches were performed using conventional telegram-style search short-string text formulations with Boolean operators as described in PubMed. Illustrative key search words were bacteria, mold, fungi, yeast, tobacco, smoke, endotoxin, mycotoxin, cured, fermented, lipopolysaccharide, aflatoxin, and microbiology. We also used unique search words, such as author's name, project designation, report codes, cigarette brands, and Bates number. The references cited in the retrieved literature were reviewed to identify other topic-specific writings Table 1.

## 7. Tobacco-Associated Chronic Inflammation

Chronic inflammation is associated with malignant transformation, tumor growth, and, possibly, tumor metastasis, reviewed in [44–52]. Examples of the association of cancer with chronic inflammation include (a) lung cancer and cigarette smoke (aerosol), (b) malignant mesothelioma and asbestos (fibers), (c) stomach cancer and *H. Pylor*i (bacteria), (d) malignant melanoma and ultraviolet sun light (irradiation), (e) liver cancer and aflatoxin (mycotoxin), and (f) cancer of the uterine cervix and human papilloma virus. Thus, malignancy at diverse body sites, and of various tissues, is associated with chronic inflammation provoked by assorted items that include smoke, bacteria, fibers, irradiation, toxins, and viruses.

## 8. Cigarette Smoke, Chronic Inflammation, and Impaired Immunity

Cigarette smoke is known to induce chronic inflammation of the lung [53–60]. More recently, a substantial body of information has been obtained to suggest that long-term cigarette smoking may not only have an adverse effect of systemic immunity but also skews both innate and adaptive immune responses [61–65].

## 9. Study Rationale: Evidence-Based Health Risks of Tobacco-Associated Microbes

Concern has been expressed by many investigators that microorganisms on cured tobacco might represent a health risk. By way of example, in 1968, Wood [66], a scientist at the British American Tobacco Company, wrote a 37-page report addressing the possible transfer of viable microorganisms into mainstream smoke. In this internal document, he notes that cured tobacco, of various types, has long been known to contain bacterial spores. Likewise, Wood [66] and others [23] have addressed the possibility that tobacco-associated mold may also represent a health hazard to smokers. Support for this concern was derived in part from a paper published in *Science* by Forgacs and Carll two years previously in which they reported the identification of toxic fungi in tobacco [23]. In the *Science* paper, the investigators exposed mice to smoke from fungally contaminated hay. The mice developed pulmonary emphysema and other pathological conditions; in contrast, mice exposed to smoke from sterile, uninoculated hay remained normal clinically. In a letter to the Associate Scientific Director of the Council for Tobacco Research, dated 1964, Forgacs, with more than 16 years of research experience as a mycologist, states that he had examined mycologically a number of tobacco products, including cigarettes that had been purchased on the open market [67]. Forgacs observed that the tobacco of all cigarettes contained fungal mycelia and spores [67]. In part, the origin of his health concern is based upon the knowledge of (a) widespread fungal contamination of tobacco products, (b) heat stability of the mycotoxins; (c) known animal toxicity, (d) reasonable assumption that some of the fungi are carcinogenic, and (e) potency at low doses, see also [68]. 

Wood argues that

“[W]hile it is quite impossible to deduce, from this (mouse) experiment, the likely effect of smoke from a cigarette containing fungally contaminated tobacco, the implications are sufficiently important to warrant some consideration of the role which micro-organisms may play with regard to smoke toxicity. For instance, it is possible that viable spores might be transferred to mainstream smoke and thus enter the lungs; pathogenic species, even in small numbers, could clearly have harmful effects, while very large number of otherwise harmless micro-organisms might lead to a significant concentration of genetic material. Alternatively, during the vegetative stage of their residence on tobacco the micro-organisms might produce toxins which could transfer direct to smoke or metabolites which on burning could give toxic smoke constituents.”

The report by Wood also describes some preliminary experiments which were undertaken to show whether bacterial or fungal spores could transfer into tobacco smoke. Two schemes were used to trap the cigarette smoke; these were a test tube bubbler and a micropore filter. These samples from the bubbler and the filter were tested for the growth of microorganisms. Growth of microbes was observed; however, technical problems were encountered including poor reproducibility and smoke toxicity. The results were inconclusive. Our search for subsequent studies by Wood addressing this subject failed to identify subsequent experiments or published reports. Studies by Slutzker et al. were negative [69]. In 1967, Curby reported to The Council for Tobacco Research the results of comparative studies that he had undertaken to determine the microbiological activity in the smoke from filter and nonfilter cigarettes. Different popular brands of cigarettes were obtained from local vendors in Brookline, Mass, USA. Comparative analyses were made of bacteria released from cigarettes that had been “cold smoked” (not lit) or smoked in the usual manner (lit). The tobacco smoke collection system was tested for sterility by means of conventional microbiology culture procedure and by means of electronic analyses of particle size and number. Viable bacteria were identified in the smoke from all cigarettes tested. The number of liberated organisms was much greater when the cigarette was burning [24, 25]. 

Before profiling more recent studies, a brief overview is warranted of what many internal documents of the tobacco industry have entitled the “*Microbiology of Tobacco.*”

## 10. Microbiology of Tobacco

The *“Microbiology of Tobacco” *has been the focus of many studies. It was not surprising to learn from our paper that most of all the major tobacco companies have studied this issue for many years. Listed below are varying topics addressing bacteria, mold, and mycotoxins in tobacco and references

- chemical and microbiological changes during curing [16, 19, 70–75],
- bacteria in cigarettes; product comparison (also, see below) [17, 76–79],
- databases of tobacco microbes [33, 40, 80],
- tobacco microbe control [81],
- microflora community of tobacco [82–88],
- quantitative studies of tobacco microflora [89–91],
- growth of mold in stored tobacco [26, 92],
- growth of *Aspergillus* from tobacco [93–95],
- microbial degradation of nicotine [18, 96],
- examination of cigarettes from mold-damaged and nondamaged tobacco [97],
- isolation of viable fungi from snuff [98],
- sterilization/treatment to remove NNK [37, 99–105],
- removal of harmful toxins on tobacco [35, 95],
- inhibiting mycotoxin production [106],
- microbiology of cigarettes, pipes, cigars, and snuff [27–30, 107–111].

From about the early 1970s, extensive research was conducted on the *Microbiology of Tobacco. *Many reports reflected the interest of the major tobacco companies. These studies sought to identify different bacteria and molds and to count the number of colony-forming units (CFU) during processing. The number of bacteria and molds present in green, freshly harvested tobacco was compared to that of various stages of curing, fermentation, and long-term storage. In many cases, more than one million bacteria were found in a gram of tobacco (a 100 mm cigarette has about 0.9 grams of tobacco). Comparative studies included various types of tobacco (Bright and Burley) and different curing methods (field versus flue cured). In these studies, profiles were established for leaves of the different types of tobacco that had been picked from various positions of the plant. Diverse environmental conditions were evaluated, and these included variations in temperature and moisture. Analyses were made of the number of bacteria in popular brand cigarettes. In many instances, the number of bacteria of a particular company's brand was compared to brands marketed by competitors. In addition to cigarettes, studies were performed for cigars and snuff. Considerable effort was devoted to defining procedures for the sterilization of tobacco to reduce or prevent the growth of mold. The methods used included (a) washing methods using various solutions (bleach), (b) irradiation with microwave, ultraviolet light, and gamma radiation, (c) exposure to various gases, and (d) treatment with different antibacterial and antifungal agents (antibiotics). One scheme was to destroy all of the bacteria on freshly harvested green tobacco leaves and then seed the leaves for fermentation using selected colonies from in-house batch-scale production. Quality control of the tobacco was important as high levels of mold produced an unacceptable “off-taste.”

## 11. Pathogenic Bacteria of Chewing Tobacco

Studies have been conducted by investigators of the tobacco industry (see above) and health community to address the potential of bacteria, molds, yeast, and microbial toxins found in different types of smokeless tobacco (snuff, snus, and long cut) [20, 26, 31, 43, 112, 113]. 

In 1951, Dynert published in *The New England Journal of Medicine* a case report of a patient with chronic bronchitis. *Pseudomonas aeruginosa, *often colonized in COPD patients, and a few colonies of *Staphylococcus aureus* were identified in bacteriological examinations of the subject's sputum [20]. The patient used snuff, and it was theorized that the snuff may have been the source of the pathogens. A study was then undertaken of 22 samples of previously unopened packs of snuff. The following microorganisms were grown from more than 50% of the snuff samples: *Bacillus rubitilles*, *Staphylococcus aureus* (coagulase positive), *Staphylococcus albus* (coagulase positive), *Pseudomonas aeruginosa*, *Staphylococcus aureus* (coagulase negative), and *Staphylococcus albus* (coagulase negative). 

In 1991, Varma reported the isolation of nine species of *Aspergillus* in stored leaves of chewing tobacco [112]. Approximately 18 of the Aspergilli were found to be mycotoxigenic. All aflatoxigenic strains of *A. flavus* produced aflatoxin B_1_. Patulin and ochratoxin were produced by *A. ochraceus*. Sterigmatocystin was produced by three different strains. 

Warke [103] studied the microbiological quality of chewable, often sweet, tobacco mixes known as “Gutka” used by millions of children and adults in India where it is made and often exported. Of the 15 samples studied, all contained aflatoxins B_1_, B_2_, and G_2_. Samples exposed to ^60^Co radiation displayed a marked reduction of viable CFU. Sterilization of tobacco in the manufacturing has been described in US Patents [105]. 

In 1992, Rubenstein reported the identification of large number (>10^6^ CFU) of a *Bacillus* species in chewing tobacco sold in the USA [31]. Supernatants of the cultured bacteria evoked a plasma exudate in studies in which the supernatant was instilled into an intact hamster cheek pouch.

## 12. Pathogenic Bacteria of Cigarettes

Some bacteria grow in unique microenvironments, and some are difficult to grow using traditional broth- and agar-based methods. This technical difficulty may also apply to growing bacteria that have adapted to unique conditions that develop during the curing and fermentation of tobacco. Accordingly, it is believed that conventional methods may not accurately define the microflora of diverse tobacco products [43, 113]. Consequently, there may be an incomplete understanding of the bacterial diversity in the tobacco of cigarettes and also the impact these microbes and microbial toxins may impose on the smoker [113]. 

Recently, the bacterial metagenomic of cigarettes were characterized using a 16S rRNA-based taxonomic microassay as well as traditional cloning and sequencing methods. The brands included Camel, Marlboro, Kool, and Lucky Strike. The results of this study showed that the number of microorganisms in cigarettes may be as vast as the number of chemicals in these products. Fifteen classes of bacteria were identified [113]. Particularly noteworthy was the identification of a broad range of potentially pathogenic microorganisms detected. More than 90% of the tobacco samples from the cigarettes contained *Actinetobacter*, *Bacillus*, *Burkholderia*, *Closteridium*, *Klebsiella*, *Pseudomonas aerogenosa*, and *Serratia*. Other bacteria that are known to be potentially pathogenic to humans and detected using the metagenomic technology were *Campylobacter*, *Enterococcus*, *Proteus*, and *Staphylococcus *[113]. 

Reported also in 2010 were the results of an investigation of the diversities of unaged and flue-cured tobacco leaves using a 16S rRNA sequence analysis scheme [43]. 

Others have reported the identification of potentially pathogenic bacteria in commercial cigarettes. One study was undertaken to assess the bacterial diversity of cigarettes that were thought to be linked to severe pneumonitis in US military personnel deployed in Operation Iraqi Freedom [38]. Eight species of *Bacillus*, including five new species, and one new species of *Kurthia* were isolated from the cigarettes. Some of these species have been identified elsewhere to cause hypersensitivity pneumonitis and other respiratory syndromes [38]. This study was of particular interest to many because the cigarettes were made in Iraq and not manufactured by a major tobacco company. Undertaking this investigation, the question arose as to whether the cigarettes that had been purchased by soldiers from street vendors had been intentionally altered by adding pathogenic bacteria and/or mold. This theory was disproven. 

Another study was conducted by a group of investigators in Sweden who characterized the bacterial and fungal community in warehouse tobacco [41]. 

We have reported previously the establishment of a novel bioassay which showed that bacteria were grown routinely from a single flake of tobacco that had been placed on the surface of a sheep blood agar plate [42]. Of eight popular brands of cigarettes, bacteria grew from almost all (>90%) of the flakes. Similarly, bacteria were grown from a single flake, and also with a high frequency, from tobacco that had been retrieved from cigar filler and from smokeless tobacco (snus, snuff, and long cut). Some bacteria induced hemolysis of the blood in the agar dishes. The destruction of the red blood cells was readily visible as a yellow zone surrounding a single tobacco flake. Expanding studies documented the hemolysis of human blood in agar or nutrient broth cultures. Thus, as discussed later, bacteria could be carried deep into the respiratory tract by a single tobacco flake sucked from the cut surface of a cigarette filter and transported into the bolus of smoke that is inhaled deep into the lung. A single tobacco flake may be envisioned as a matrix for delivering diverse bacteria into the respiratory tract of an immunologically compromised long-term smoker.

## 13. Cigarettes with Mold

Mold has been identified in the tobacco of popular brand cigarettes, and concern has been raised as to the propensity of these microbes as a health risk to the smoker. Presented herein is a partial listing of papers that have identified mold in cigarettes [78, 114–116] and in marijuana [116]. 

As early as 1971, Papavassiliou and coworkers concluded that “[C]igarettes are contaminated with various fungi.” They studied cigarettes that were manufactured in the USA, Canada, England, France, Belgium, Germany, Jordan, and Egypt. Hundreds of strains of fungi were isolated. The Greek scientists demonstrate that the most prominent fungi were *Aspergillus* (28 strains from Greek cigarettes and 35 strains from other countries). They raised the question as of the association of the fungi with allergies but commented that this issue has not been resolved [114]. 

In 1983, Kurup and colleagues reported the identification of allergenic fungi in smoking materials and discussed the health implications of their findings [115]. Concern has been expressed as to the health risks associated with mold in cigarettes.

Writing in the Journal of the American Medical Association, Verweij et al. addressed the propensity of heath risks associated with fungal contaminates of tobacco and marijuana [116]. They concluded that “[A]ll cigarette brands tested (*N* = 14 brands) had some degree of fungal contamination, although not every cigarette was found to have a positive culture.”

## 14. Transfer of Tobacco Flake to Mainstream Smoke

The filter of a cigarette is often contaminated with loose tobacco flakes, tobacco fines, and tobacco dust. In one examination, the filters of 11 brands of cigarettes were examined in freshly opened packs. For all brands, cigarettes were observed with tobacco flakes on the filter. Examination of the filters with the naked eye showed that 127 of 208 (61.1%) of the filter had tobacco particles [42]. The release of tobacco flakes into mainstream smoke has been described previously [21, 22]. 

The tobacco flakes that contaminate the filter arise from tobacco that escapes from the nonfilter, sometimes called the distal, end of the cigarette. Most probably the flakes are jarred loose during manufacturing, shipment, and daily transportation, especially in a pack in which more than one-half of the cigarettes have been used [117, 118].

The release of flakes from the cut surface can readily be demonstrated by comparing the cut surface of the filter before and after smoking the first puff. The single flake may be viewed as a matrix for carrying bacterial and fungal agents in mainstream tobacco smoke. Thus, the burning of the tobacco during cigarette smoking does not exclude the exposure to tobacco-associated microbes and microbial toxins. 

Bacteria are also released from the barrel of the cigarette. This was demonstrated in investigations in which a cigarette was rolled over the surface of a nutrient agar dish.

## 15. Endotoxin (LPS) in Mainstream and Sidestream Tobacco Smoke

In 1999, Hasday and his colleagues reported the identification of bacterial endotoxin as an active component in cigarette tobacco and cigarette smoke [34]. The authors showed that the dose of LPS delivered from smoking one pack of cigarettes was comparable to that of the LPS that had been previously shown to be associated with adverse health effects in cotton textile workers. With the knowledge that LPS is one of the most potent inflammation-inducing agents, the work by Hasday attracted considerable attention, reviewed in [32]. In 2004, Larsson et al. reported that they were able to demonstrate unequivocally that high levels of LPS are inhaled during active cigarette smoking and, more importantly, that environmental tobacco smoke may involve inhalation of amounts of endotoxin that are dramatically greater than those existing in indoor environments free from tobacco smoke [36]. In 2006, these findings were confirmed and extended [39]. Particularly notable is that studies of Larsson and colleagues used a mass-spectrometry-based assay that circumvents the problems often associated with the biologically based LPS assay.

## 16. Analysis of Findings and Policy Recommendations

The results of this literature review have documented that the tobacco microflora has been the subject of many studies by investigators of tobacco industry and academic communities. During the last 50 years, there has been an imbalance, however, in the attention devoted to addressing the identification and propensity of the harm of tobacco- and tobacco-smoke-associated chemicals and in the attention devoted to characterizing microbes and microbial-derived factors. 

Ample information has accumulated to suggest that microbes and microbial-derived factors may contribute to the health risks of smoking and smokeless tobacco products. Moreover, the microbes may facilitate microbial colonization of the mouth and airway, the induction of chronic inflammation through the activation of diverse leukocyte subsets, alteration of the tissue microenvironment, and microbial-toxin-induced pathologies. The current health concerns recently expressed by investigators of various disciplines, and with different research interests, in peer-reviewed published research articles are reasonable and validate that additional investigation of the microbiology of tobacco is warranted. The findings reported herein relate to National Tobacco Control Policy and specifically FDA Regulation of Tobacco Products [119]. 

Based upon the information obtained in this paper, we recommend the following for consideration and possible regulatory action.

1. Tobacco products should be assessed with the knowledge that they contain bacteria, mold, and microbial toxins.
  - In this context, the designation of tobacco products is to include conventional and novel products that contain tobacco, including items which are smoking and smokeless tobacco articles.
  - National and international registries of known human carcinogens should not be used as the sole criteria for assessing tobacco-associated human health risks. Any and all tobacco-associated agents that induce any human pathology should be included in risk assessments.
  - Tobacco in smoking and smokeless tobacco articles should be assessed for their propensity to induce chronic inflammation. Chronic inflammation is known to be induced by diverse bacteria (Gram positive and Gram negative) and fungi, living or dead, whole or fragmented, and intracellular and membrane components. Chronic inflammation is known also to be induced by diverse toxins of bacteria and/or fungi including, but not limited to, endotoxins, exotoxins, and mycotoxins.
  - Chronic inflammation associated with bacteria, fungi, and microbial toxins of tobacco products should include inflammation of any and all target sites, including tissues of the mouth, nasopharynx, and lung.
  - In addition to chronic inflammation, harm of microbial elements of tobacco should be assessed in the context of other known tobacco-associated diseases, including chronic obstructive pulmonary disease, asthma, bronchitis, and alveolar hypersensitivity.
2. Tobacco-specific nitrosamines (NNK) are human carcinogens that are present in mainstream smoke, sidestream smoke, and smokeless products. NNKs arise primarily from the microbial degradation of nicotine in tobacco. Different technologies have proven effective in preventing the formation of NNKs. It is recommended that these technologies be implemented and that guidelines for tobacco articles be established for reduced NNK-products.
3. The criteria, protocols, and procedures used by the FDA in the assessment of harm associated with mycotoxins in food products should be applied to loose leaf tobacco, smoking tobacco products, and smokeless tobacco articles. Mycotoxin action levels should be established to provide an adequate margin of safety to protect human tobacco users.

## Figures and Tables

**Table 1:** History of investigations of microbes and microbial toxins in tobacco and tobacco products.

1896 [16]	Results are reported for studies that were undertaken to characterize the microbes of tobacco before and during tobacco fermentation.

1899 [17]	German bacteriologist H. E. Suchsland announces that the delicate aroma and subtle shades of flavor which affect the palate of the smoker are not due to the tobacco but are attributed to the microbes which aid in the process of tobacco fermentation. A patent based upon this observation was submitted, presumably to improve the poor quality of German tobacco by adding to the harvested tobacco leaves bacteria that he had isolated and grown in his laboratory from high-quality West Indian tobacco.

1954 [18]	The microbial degradation of nicotine and nicotinic acid was reported. The morphological and physiological properties of the nicotine-decomposing bacteria were also described.

1955 [19]	W. C. Flanders of R. J. Reynolds Tobacco Company issues a 70-page report of a three-year study to determine if the number of microorganisms (bacteria and mold) changed appreciably during aging. Experiments were also conducted to determine if the recorded changes in the microbes follow the changes in the chemical components of tobaccos. These studies were continued and extended for several years.

1957 [20]	*Pseudomonas aeruginosa* and other potentially pathogenic fungi and bacteria were identified in snuff. Similar microbial isolates from a patient was the basis for the physician to theorize that some of the snuff-derived microbes may be responsible in part for chronic bronchitis.

1958 [21, 22]	The results of studies were reported that had been undertaken to characterize the deposition of cigarette smoke particles and debris released from the cigarette filter into the human respiratory tract. Popular brand cigarettes were smoked mechanically and in a manner to reflect normal smoking behavior. The studies documented that tobacco flakes and fine tobacco leaf debris were released into mainstream smoke from the cigarette filter of all brands that were tested (Tareyton, Winston, Kent, L&M, Marlboro, and Viceroy). The tobacco flakes and other particulates (filter fibers and carbon from charcoal filters) were studied by light and electron microscopy.

1966 [23]	Toxic fungi were identified in tobaccos.

1967 [24, 25]	Comparative studies were preformed for microbiological activity in the smoke of popular brand nonfiltered and filtered cigarettes that had been “cold smoked” or lit. Viable bacteria were found in the smoke of all cigarettes tested.

1972 [26]	The tobacco from different popular brands of cigarettes was analyzed for bacteria. The number of bacteria was determined on “our own” (Philip Morris) and competitive cigarette fillers. This test was run for several months and each month Viceroy, Brown & Williamson's product, always showed the lowest degree of “contaminant.” The difference between the brands was statistically significant. Brands tested included Salem, Pall Mall, Chesterfield, Kool, Kent, Viceroy, Winston, and Marlboro. The number of bacteria on Marlboro were “too numerous to count.”

1972 [27]	A 189-page report was prepared by investigators at the Brown & Williamson Tobacco Company that presents methods for the microbiological examination of tobacco and tobacco products. The writings include the description of techniques for the quantitative determination of bacteria and fungi and methods for the isolation of potentially human pathogenic microorganisms including Coliform bacteria. Also identified were *Staphylococcus aureus*, *Enterococci*, *Pseudomonas*, *Clostridium*, and *Aspergillus*.

1972 [28]	A 52-page report that describes a “contact plate method” in which a whole cigarette is rolled over the surface of the nutrient agar dish. Viable microbes that are transferred from the cigarette to the plate are illustrated. Presumably, the intent of the assay was to measure the growth of microbes that would be transferred from the cigarette paper to the hand of the smoker. Other studies showed the growth of microbes from a natural wrapper of a cigar. Also, culture methods were established for testing for coliform bacteria and for counting viable fungi in tobacco.

1972 [29]	A 346-page in-house document is produced by the British-American Tobacco Company entitled “Methods for the Microbiological Examination of Tobacco and Tobacco Products.” The authors describe the “Public Health Aspects” of smoking and smokeless tobacco products. They note that “[T]he detection of micro-organisms of health significance in tobacco products must be expected to be regarded as undesirable or even unacceptable by public agencies, regardless of whether there is proof of the significance in initiating or spreading infection in man. Therefore, it is suggested that tobacco products should be substantially free, or contain only minimal numbers, of micro-organisms of potential health significance to man which could conceivably occur on tobacco…” Suggested standards are presented for tobacco products for various bacteria and fungi, and standards that had been established for food products (fish, sausage, meat pies, cream yogurt, soft cheese, and pasteurized milk).

1991 [30]	Philip Morris characterizes the microbial population on Marlboro tobaccos throughout the processing line. Five different Marlboro Make-Your-Own tobaccos with various anti-microbial preservatives were evaluated microbiologically for mold and bacteria over time. The microflora of Marlboro raw and tobacco blends were defined for burley, oriental, flue-cured, and other tobacco types.

1992 [31]	Bacillus spores were identified in chewing tobacco sold in the USA. Broth of the culture microbes evoked plasma exudation from the oral mucosa when tested using a hamster cheek pouch assay.

1995 [32]	In an oral presentation, Hasday describes for the first time the presence of endotoxin in cigarette smoke.

1990 [33]	Scientist from Imperial tobacco (Canada) report the development of an easy-to-search database on the microbes associated with tobacco.

1999 [34]	Bacterial endotoxin was identified as an active component of cigarette smoke.

2004 [35]	A US Patent was awarded for a method and system for assay and removal of harmful toxins during the processing of tobacco products.

2004 [36]	Microbiologists in Sweden used a mass-spectrophotometry-based assay to document that tobacco smoking increased dramatically the air concentrations of endotoxin (LPS). The authors note that smoke-derived LPS may be a health risk factor associated with environmental tobacco smoke.

2004 [37]	A US Patent was assigned to Philip Morris for an “antibacterial lavage” method to treat tobacco leaves so as to eliminate or reduce bacterial endotoxins (LPS) and tobacco-specific nitrosamines that are formed during the curing process. Bacteria found on tobacco leaves were reported to be primarily Gram-negative bacteria, including pseudomonades and enterobacters. In the awarded patent, Hempling notes that bacterial endotoxins can remain as a residue on the tobacco even after the bacteria have been destroyed.

2004 [36]	The microbiological composition of tobacco products was defined using culture and chemical analysis. Tobacco smoke was analyzed chemically, and LPS was measured for tobacco leaves and cigarette tobacco.

2005 [38]	US Military publishes a report of an investigation that documents bacterial species diversity of varying brands of cigarettes made in the Middle East that were thought to be associated with illnesses of American soldiers deployed in Operation Iraqi Freedom.

2006 [39]	Cigarette smoke was identified as the source of elevated levels of endotoxin (LPS) found in indoor air.

2007 [40]	Identification of microflora on tobacco using culture-independent methods based on the amplification of microbial 16S rDNA sequences directly from the leaf surfaces. The investigators discovered also that three of five dominant bacterial species on the tobacco could not be cultivated.

2008 [41]	The microbiological composition of tobacco products was defined using culture and chemical analysis (of tobacco leaves) or chemical analysis only (tobacco and tobacco smoke). Mesophilic bacteria dominated among the bacteria in both fresh and cured tobacco leaves; however, a wide range of other bacteria, including Gram-negative bacteria, and fungi were delineated. Microbial flora was compared in studies of tobacco from cigarettes from different countries. LPS was also measured.

2008 [42]	Bacteria grown from a single flake of tobacco from all brands of smoking (cigarette, cigar, and pipe) and smokeless (snus, snuff, and long cut) tobacco products. In many instances, the bacteria from the tobacco caused hemolysis of blood in blood agar and liquid broth cultures.

2010 [43]	Twenty-seven species of bacteria were identified in an analysis of both unaged tobacco and flue-cured tobacco by 16S rRNA sequence analysis. More species (*N* = 23) were identified from the unaged flue-cured tobacco leaves than in the aging leaves (*N* = 15 species).

2010 [43]	Fifteen classes of bacteria and a broad range of potentially pathogenic organisms were detected in all cigarette samples studied. In greater than 90% of the tobacco samples, the investigators identified *Acinetobacter*, *Bacillus*, *Burkholderia*, *Clostridium*, *Klebsiella*, *Pseudomonas aeruginosa*, and *Serratia*. The bacteria were identified using a 16S rRNA-based taxonomic microarray. Cloning and sequencing were used to evaluate total bacterial diversity of four brands of cigarettes. Previous studies have shown that smoking was associated with colonization by pathogenic bacteria and an increased risk of lung infection. This study, however, was the first to show that cigarettes themselves could be the source of exposure to a wide array of potentially pathogenic microbes.

## Abbreviations

AFL-B_1:_: Aflatoxin, type B_1_
CFU: Colony forming unit
DC: Dendritic cell
IARC: International Association for Research of Cancer
IL-1*β*: Interleukin-1, beta
IL-4: Interleukin-4
LIF: Leukemia inhibitory factor
LPS: Lipopolysaccharide
LSRO: Life Science Research Organization
LTDL: Legacy Tobacco Document Library
MON: Monocyte
NTP: National Toxicology Program
OSM: Oncostatin M
PGE_2:_: Prostaglandin E2
PMN: Polymorphonuclear leukocyte
RNS: Reactive nitrogen species
ROS: Reactive oxygen species
TLR: Toll-like receptor
TNF*α*: Tumor necrosis factor, alpha.
